# Treatment of lumbar vertebra epithelioid hemangioma with radiation therapy: a case report

**DOI:** 10.1186/s13256-019-2237-y

**Published:** 2019-10-15

**Authors:** Aldis P. Siltumens, Na L. Smith, Rosalind F. Sharain, Michael G. Haddock, W. Michael Hooten

**Affiliations:** 1 Department of Anesthesiology and Perioperative Medicine, Mayo Clinic, 200 First St SW, Rochester, MN 55905 USA; 2 Department of Radiation Oncology, Mayo Clinic, Rochester, MN USA; 3 Department of Laboratory Medicine and Pathology, Mayo Clinic, Rochester, MN USA

**Keywords:** Epithelioid hemangioma, Back pain, Pain management, Radiation therapy

## Abstract

**Background:**

Although epithelioid hemangiomas involving bone have been described in previous case reports and case series, the effects of radiation therapy on vertebral epithelioid hemangioma has not been fully reported. Here we provide a case report of tumor response to radiation therapy in a young adult with a large epithelioid hemangioma involving the fourth lumbar vertebrae.

**Case presentation:**

A 27-year-old Latino man with a past medical history of type 1 diabetes and a 3-year history of low back pain presented to a hospital emergency department following acute worsening of back pain. On transfer to our tertiary medical center, he described the pain as “shock-like” which originated at the lateral aspect of his right hip and radiated down to his right knee. Paresthesia was also reported along the medial aspect of his lower right leg. Imaging included a computed tomography scan and magnetic resonance imaging which revealed fourth lumbar and right iliac lytic bone lesions. Image-guided biopsies of the lytic lesions were consistent with a diagnosis of epithelioid hemangioma and radiation therapy was recommended as the primary treatment. Our patient’s low back and leg pain were initially managed with acetaminophen, oxycodone, pregabalin, and lidocaine patch 5%. He noted improvement in pain after his third fraction of radiation. Pain intensity continued to decline and oxycodone was discontinued.

**Conclusions:**

This case report demonstrates an unusual etiology of back and leg pain in a young man and elucidates the palliative effects of radiation therapy for epithelioid hemangioma involving the lumbar spine.

## Background

Epithelioid hemangioma (EH) affecting the lumbar vertebra is an uncommon variation of a rare disease [1]. Hemangiomas are tumors typically composed of thin-walled blood vessels, of which EH is an infrequent variant with an exact incidence that is unknown [2]. EH is considered benign but can be locally aggressive [1–3]. EHs most commonly affect the integumentary system; however, several other sites have been described, including lymph nodes, lung, eye, and breast. Bone is the second most commonly affected site, but involvement of lumbar vertebra has not been widely reported [4].

Treatment of EH includes simple surveillance, surgery, and radiation. Many patients treated surgically undergo curettage, but the role of radiation therapy varies widely [1, 3]. For example, radiation therapy has been used as an adjunct to surgery but it has rarely been reported as a primary treatment for EH involving the lumbar vertebra. Here, we provide a case report of EH involving the lumbar spine treated solely with radiation therapy.

## Case presentation

A 27-year-old Latino man with a past medical history of type I diabetes and chronic low back pain of 3 years’ duration presented to the local emergency department for evaluation of acute worsening of the low back pain rendering him unable to bear weight. Computed tomography (CT) and magnetic resonance imaging (MRI) of his lumbar spine demonstrated lytic bone lesions involving the fourth lumbar (L4) vertebral body and right iliac crest. Conservative management over a period of 1 week at a local hospital failed to provide pain relief. Subsequently, he was transferred to our institution for further management.

**Fig. 1:**
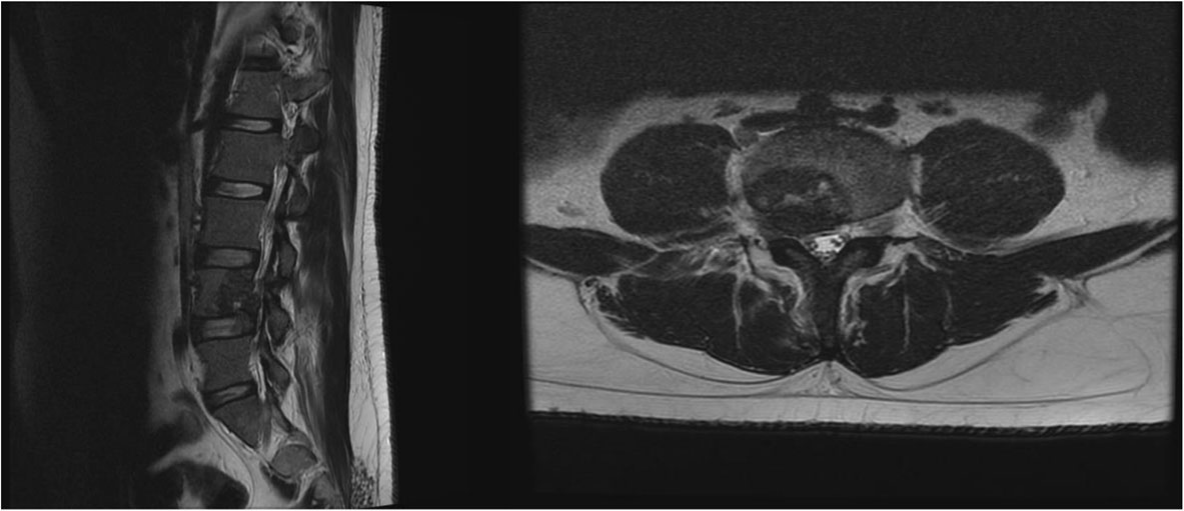
Repeat magnetic resonance imaging at our institution revealed interval increase in size of destructive lesions involving the fourth lumbar vertebral body and fourth lumbar spinous process. Enhancement along the right aspect of the cauda equina extending cephalad from the level of the epidural soft tissue extension at fourth lumbar was also noted

On review of symptoms, he described experiencing “shock-like” pain, which originated at the lateral aspect of his right hip with radiation to his right knee. Pain was accompanied by paresthesia along the medial aspect of his right lower leg and worsened with lumbar spine flexion and extension. He rated the pain at 4 using the Numeric Rating Scale (NRS; 0 = no pain, 10 = worst possible pain). He was unable to maintain an upright position due to pain. Chronic difficulties with initiation of urination and constipation were also reported. The right lower extremity pain was reproduced on physical examination by right straight leg raising test to 30 degrees above the horizontal and left straight leg raising test to 45 degrees. Repeat lumbar spine MRI with and without contrast at our institution revealed interval increase in size of the indeterminate destructive lesions at the L4 vertebral body with pathologic fracture, the L4 spinous process, and the right iliac bone, compared to the outside MRI of his lumbar spine from 10 days prior. New enhancement along the right aspect of the cauda equina extending cephalad from L4 was also evident (Fig. 1).

While awaiting further assessments, he was started on scheduled orally administered acetaminophen 1000 mg every 6 hours, scheduled orally administered pregabalin 150 mg twice daily, 5% lidocaine patch, and orally administered oxycodone 10 mg every 4 hours as needed. However, these medications provided inadequate pain relief, and he still could not maintain an upright position.

**Fig. 2:**
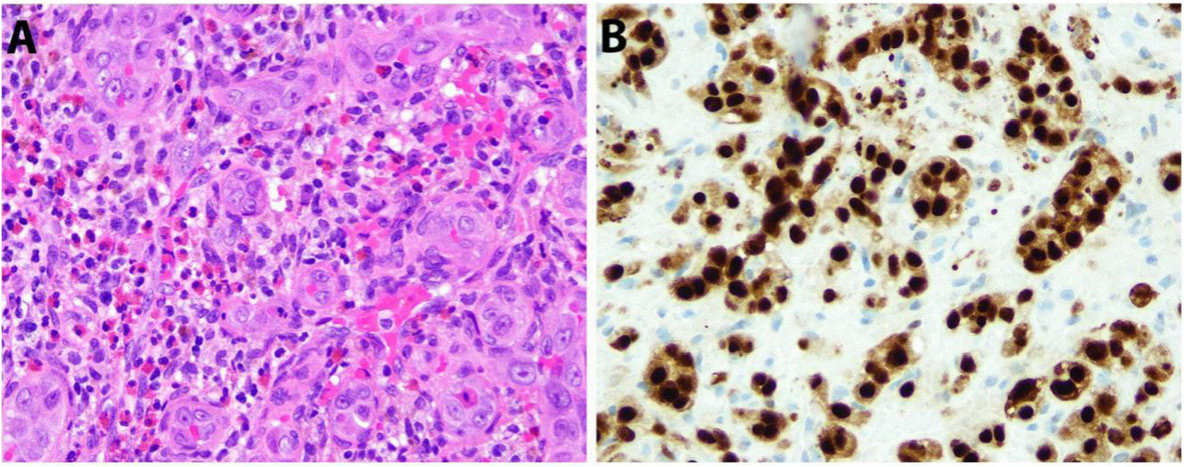
The bone biopsy revealed an epithelioid hemangioma (**a**, hematoxylin and eosin, × 40) characterized by a proliferation of well-formed vessels with epithelioid endothelial cells and a prominent inflammatory stroma with numerous eosinophils. A FosB immunostain showed strong nuclear immunoreactivity within the endothelial cells (**b**, × 40), supporting the morphologic impression

A core biopsy of the right iliac bone lesion revealed a vascular proliferation composed predominantly of well-formed capillary channels lined by epithelioid endothelial cells that appeared to protrude into the vascular lumina (Fig. 2). These lesional cells contained rounded-to-lobated nuclei and abundant deeply eosinophilic cytoplasm. The background stroma contained a prominent inflammatory infiltrate composed of eosinophils, lymphocytes, and scattered plasma cells. By immunohistochemistry, the lesion was positive for FosB, which is expressed in a subset of EHs. These histologic and immunohistochemical findings confirmed the diagnosis of an EH.

**Fig. 3:**
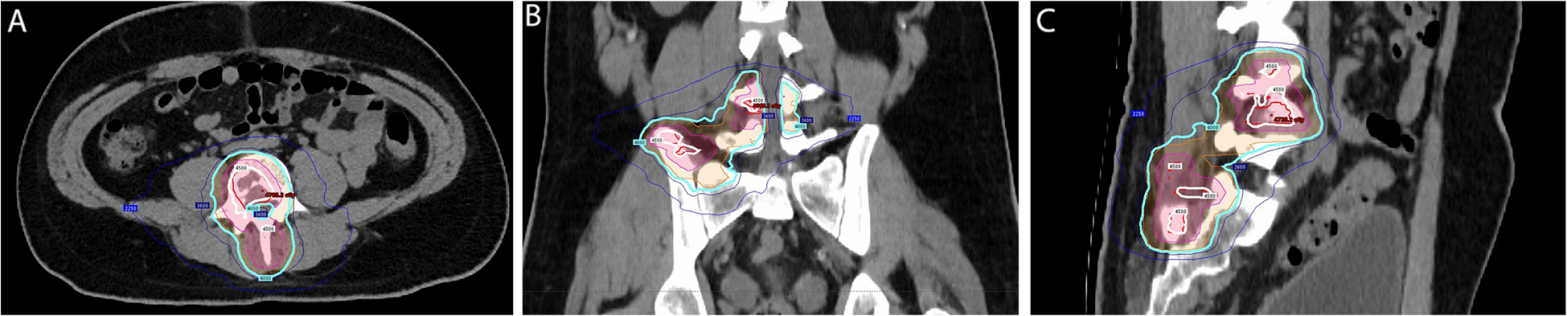
Intensity-modulated radiotherapy plan was designed with two planning target volumes: planning target volume 4500 (*red*) and planning target volume 4000 (*orange*). Isodose line: 4500 cGy (*white*), 4000 cGy (*cyan*). Representative views of the treatment plan: **a** axial, **b** sagittal, **c** coronal

Given the morbidity of a surgical approach, the decision was made to treat the tumor with radiotherapy alone. A photon intensity-modulated radiotherapy (IMRT) arc therapy plan was generated with two planning target volumes (PTVs): a high-dose volume (PTV 4500), which comprised the gross tumor volume (GTV), with a prescription dose of 45 Gy in 10 fractions; and a low-dose volume (PTV 4000), which comprised the volume of a 1 cm radial expansion from the GTV, with a prescription dose of 40 Gy in 10 fractions (Fig. 3). A low-dose clinical target volume (CTV) 4000 was generated with a 0.5 cm radial expansion of GTV. For PTV 4500, the finalized treatment plan achieved a D95% (the minimum dose covering 95% of the target volume) of 100.5% of the prescription dose, with a V100% (the minimum target volume receiving 100% of prescription dose) of 97% and a V95% of 99.6%, and V115% (the maximum target volume receiving 115% of the prescription dose) of 0 ml. A V95% of 99.9% and a V100% of 99.5% were achieved for PTV 4000 and CTV 4000, respectively. The dose-limiting structures included: cauda equina, V30Gy (the maximum target volume receiving 30 Gy) = 6.3 ml and D0.03ml (the maximum dose received by 0.03 ml of the target volume) = 39.82 Gy; small bowel, V19.5Gy = 4.97 ml, D0.03ml = 26.04 Gy; colon, D0.03ml = 28.97 Gy; and bladder wall D0.03 ml = 9.31 Gy.

The radiotherapy was delivered in ten consecutive daily treatments starting on day 9 of an 18-day hospital stay. Our patient reported his worst pain of 7/10 on day 10, and he had improvement in both pain and paresthesia on day 11. On day 12, he had resolution of urinary retention and was able to ambulate. He was able to discontinue narcotic analgesics on day 15. He was subsequently discharged with pregabalin, acetaminophen, ibuprofen, and lidocaine patch for pain management on day 18 at the completion of the 10-day course of radiotherapy with no adverse effects from radiation therapy.

**Fig. 4:**
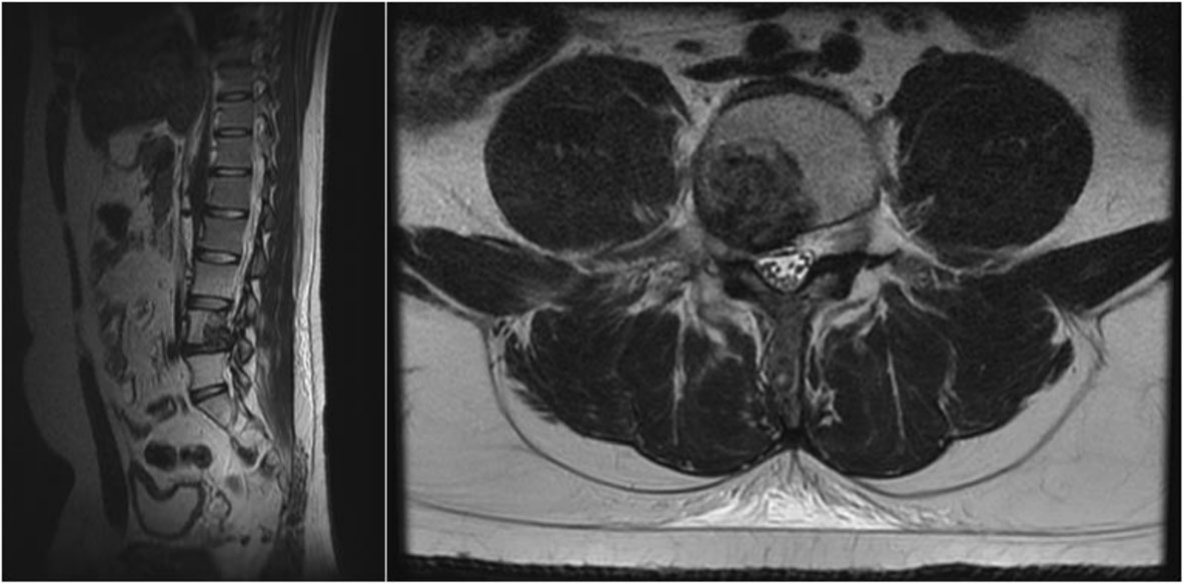
Follow-up magnetic resonance imaging at 3 months demonstrated interval improvement including post-radiation changes and decreased enhancement

At a 3-month follow-up after completing radiotherapy, he reported mild right knee pain; however, he was able to ambulate with conservative management. MRI of his lumbar spine with and without contrast demonstrated the lesions at L4 vertebral body, L4 spinous process, and right iliac bone were stable in size with decreased enhancement, and there was less enhancement and tissue within the epidural space and right neural foramen (Fig. 4).

## Discussion

EH is a rare benign vascular tumor that uncommonly involves vertebrae. Some controversy exists regarding whether these lesions are truly neoplastic or are simply reactive vascular proliferations. However, recent genetic studies support the neoplastic nature of at least a subset of EH, which have been found to harbor *FOSB* gene rearrangements [5]. Interestingly, *FOSB* gene rearrangements are more prevalent in EH affecting bone compared to other involved sites [6]. This report exemplifies several distinct aspects of EH with vertebral destruction.

Detailed descriptions of the clinical presentation of EH are limited, ranging from asymptomatic with diagnosis at autopsy to localized pain with multiple neurological deficits [7–9]. In these studies, the duration of pain prior to diagnosis varied widely from 1 to 2 weeks to over 10 years [8]. Our patient had a 3-year history of chronic low back pain with chronic urinary retention, and he presented with acute onset of worsening of the pain without worsening of urinary function or development of new neurologic deficits.

Among cases of EH arising in bone, only 16% occurred in the vertebra within the largest series of 50 cases reported to date [10]. Treatment options include combinations of biopsy, curettage, en bloc excision, preoperative embolization, and postoperative radiotherapy. Postoperative radiotherapy of 45–50 Gy with long-term local control and pain relief has been reported [3, 7]. However, the fractionation of radiotherapy was not described. Although radiation therapy has been reported to be effective in providing pain control and tumor shrinkage in large unresected hemangiomata [9], there is little information regarding radiation effectiveness in EH. To the best of our knowledge, only one patient with EH affecting vertebrae treated with radiotherapy alone has been described [4]. This case report represents the second case, and our patient was treated with a total dose 45 Gy delivered in 10 fractions to the GTV and 40 Gy to the area of involved bone. With this regimen, according to the linear-quadratic model, assuming an α/β ratio of 10, the 40 Gy volume received a biologically effective dose (BED) of 56 Gy_10_ and equivalent dose in 2 Gy fractions (EQD2) of 47 Gy. Assuming an α/β ratio of 3, the 40 Gy volume BED was 93 Gy_10_ and EQD2 was 56 Gy. Radiotherapy provided early-onset pain relief which was clinically evident after the third treatment. Improvements in pain were progressive through the course of treatment, and at the time of hospital dismissal the majority of our patient’s pain scores were rated as 0/10. However, the follow-up of our patient is short (3 months) compared to previous reports [3].

## Conclusions

This case report detailed the presentation, diagnosis, and treatment of a case of EH involving the lumbar spine and iliac bone with debilitating pain being the predominant symptom. The short-term outcome of this patient indicates hypofractionated radiotherapy alone is effective in providing local disease control and pain relief.

## Data Availability

Data sharing is not applicable to this article as no datasets were generated or analyzed during the current study.

## Abbreviations

BED: Biologically effective dose
CTV: Clinical target volume
EH: Epithelioid hemangioma
EQD2: Equivalent dose in 2 Gy fractions
GTV: Gross tumor volume
L4: Fourth lumbar
MRI: Magnetic resonance imaging
PTV: Planning target volume
